# Improved lung cancer classification by employing diverse molecular features of microRNAs

**DOI:** 10.1016/j.heliyon.2024.e26081

**Published:** 2024-02-10

**Authors:** Shiyong Guo, Chunyi Mao, Jun Peng, Shaohui Xie, Jun Yang, Wenping Xie, Wanran Li, Huaide Yang, Hao Guo, Zexuan Zhu, Yun Zheng

**Affiliations:** a State Key Laboratory of Primate Biomedical Research; Institute of Primate Translational Medicine, Kunming University of Science and Technology, Kunming, Yunnan 650500, China; b College of Horticulture and Landscape, Yunnan Agricultural University, Kunming, Yunnan, 650201, China; c College of Big Data, Yunnan Agricultural University, Kunming, Yunnan, 650201, China; d Department of Thoracic Surgery, The First People's Hospital of Yunnan Province, i.e., The Affiliated Hospital of Kunming University of Science and Technology, Kunming, Yunnan 650032, China; e College of Computer Science and Software Engineering, Shenzhen University, Shenzhen, Guangdong 518060, China; f School of Criminal Investigation, Yunnan Police College, Kunming, Yunnan 650223, China; g Department of Cardiology, The First Affiliated Hospital of Kunming Medical University, Kunming, Yunnan 650032, China; h National Engineering Laboratory for Big Data System Computing Technology, Shenzhen University, Shenzhen, Guangdong 518060, China

**Keywords:** Lung adenocarcinoma, MicroRNA (miRNA), MiRNA editing, Classification, Feature selection

## Abstract

MiRNAs are edited or modified in multiple ways during their biogenesis pathways. It was reported that miRNA editing was deregulated in tumors, suggesting the potential value of miRNA editing in cancer classification. Here we extracted three types of miRNA features from 395 LUAD and control samples, including the abundances of original miRNAs, the abundances of edited miRNAs, and the editing levels of miRNA editing sites. Our results show that eight classification algorithms selected generally had better performances on combined features than on the abundances of miRNAs or editing features of miRNAs alone. One feature selection algorithm, i.e., the DFL algorithm, selected only three features, i.e., the frequencies of hsa-miR-135b-5p, hsa-miR-210-3p and hsa-mir-182_48u (an edited miRNA), from 316 training samples. Seven classification algorithms achieved 100% accuracies on these three features for 79 independent testing samples. These results indicate that the additional information of miRNA editing is useful in improving the classification of LUAD samples.

## Highlights

• The editing features of miRNAs are used to construct cancer classification models for the first time.
• Three types of molecular features of miRNAs are generated for 5 blocks of LUAD and control samples.
• The ComBat-Seq algorithm is used to remove the batch effects of the data sets used.
• hsa-miR-135b-5p, hsa-miR-201-3p and hsa-mir-182_48u (an edited miRNA) are selected from the training data set.
• Seven algorithms achieve 100% accuracies on the three selected features for independent testing data.

## 1 Introduction

Lung cancer is the leading cause of cancer death worldwide, with an estimated 1.8 million deaths (18%) [1]. Nearly a quarter of all cancer deaths are caused by lung cancer, with 82% directly attributable to smoking [2]. Lung adenocarcinoma (LUAD) is the most common histologic subtype of non-small cell lung cancer (NSCLC), occurring in both females (smokers and non-smokers) and non-smoking males [3]. Most adenocarcinomas occurred initially in the outer regions of the lungs and had a tendency to spread to the lymph nodes and elsewhere [3]. Despite advances in diagnosis and treatment, mortality from lung cancer continues to rise, with the highest of all cancer types [3], [1].

MicroRNAs (miRNAs) are a class of small single-stranded non-coding RNAs of approximately 22 nucleotides (22 nt) in length [4], which act as endogenous negative regulators of genes at the post-transcriptional levels [5], [6], [7]. MiRNAs are powerful regulators of a variety of cellular activities, including cell growth, differentiation, development and apoptosis [8]. There is growing evidence that miRNAs are severely dysregulated in human cancers, including LUAD, and may act as either oncogenes or tumor suppressors [9], [10].

RNA editing is a widespread post-transcriptional gene regulation mechanism in animals, and is a specific modification of RNA sequences [11], [12]. A-to-I editing of RNA is catalyzed by ADAR1 or ADAR2 [13]. A-to-I editing of miRNAs is widely reported [14], [15], [16], [17], [18], [19], [20], [21], [22], [23], [24], [25]. C-to-U editing is performed by APOBEC proteins [26], [27], [24], [28] and is reported in some miRNAs [29], [30], [31]. MiRNAs are modified at 3' end to add non-templated nucleotides [32], such as U or A, which are also named as 3' modifications or 3'-U adding [33], [34] and 3'-A adding [35] events. The 3'-addition is a highly conserved mechanism in animals and plants, and performed by terminal nucleotidyltransferases (TENTs) [36], [35], [37], [32].

Accumulated evidence indicate that RNA editing is deregulated and relevant in cancer. For examples, Wang et al. [38] analyzed 8595 samples of 20 cancer types and found 19 A-to-I editing sites correlated extensively with tumor subtype, disease stage, and patient survival time. In another pan-cancer analysis, Pinto et al. [39] found the editing levels of these A-to-I editing sites are generally reduced in tumors, which allows an accurate classification of normal/tumor samples. Warnefors et al. [40] also found that editing levels of conserved A-to-I editing sites in miRNAs generally had lower editing levels in cancerous tissues. Other types of editing play roles in cancer too. For example, in glioblastoma, miR-324-3p are upregulated along with the upregulation of TUT4/7, while miR-324-5p is downregulated, which promotes the proliferation of tumor cells [34]. Recently, Xie et al. [41] found that many miRNA editing sites of different types had different editing levels in three subtypes of leukemia. Liu et al. [42] identified 122 miRNA editing sites of different types that were differentially edited in clear cell renal cell carcinoma. In neurological diseases [43], [44], [31], there are also hundreds of miRNA editing sites with different editing levels compared to controls.

Some studies performed cancer classification or clustering based on miRNA abundances. For examples, Lu et al. [45] observed that most miRNAs had lower expression levels in tumor samples than in normal samples and found that classifiers based on miRNA expression levels could correctly classify 12 mouse samples and classify poorly differentiated samples with accuracy of 70.6%. Leidinger et al. extracted 5 miRNAs from 74 NSCLC (non-small cell lung cancer) samples and 20 normal control samples and obtained an accuracy of 94.5% [46]. Hung et al. [47] extracted three miRNAs from 122 gastric cancer serum samples and 70 normal serum control samples, and built classification models with accuracy of 93%. Xia et al. used 25 miRNA features extracted from 502 head and neck squamous cell carcinoma patients and 44 adjacent-tumor samples to build a classification model, which had an accuracy of 74.85% [48]. Umu et al. [49] used 202 miRNAs in serum of 535 lung cancer samples and 263 normal control samples to build classification models which reached accuracies around 70%. Inagaki et al. [50] recently used serum miRNA sequencing profiles to construct an accurate classification model for lung cancer classification with sensitivity of 89.2% and specificity of 95.9%. As reported previously [38], [39], [40], miRNA editing sites have different editing levels in cancerous tissues, suggesting that miRNA editing is useful to improve the performance of classification models. More importantly, Distefano et al. [51] recently reported that clustering of cancer samples could be improved by integrating the abundances of original and edited miRNAs (or isomiRs). However, as listed in Table S1, existing studies of miRNA-based cancer classifications only used the abundances of miRNAs, detected with small RNA sequencing, microarray or qRT-PCR based methods, without considering editing of miRNAs.

Furthermore, most of the existing studies (as summarized in Table S1) used sequencing profiles generated in the same batches and with the same sequencing instruments. The classification models proposed in these work may be incompatible with the data generated in other studies or batches due to batch effects, as noticed previously [52]. For examples, Dong et al. [53] constructed a classification model with data of TCGA as training data, and two different sets as testing data, and acquired an unsatisfactory accuracy 87.5%. Some studies also tried to solve the batch effects. For examples, Lopez-Rincon et al. [54] build 8 different models on 100 miRNAs selected from sequencing based TCGA data, and validated these models on 14 GEO data sets generated with microarray with complex normalization procedures. Two of the 8 models, performed well on the selected data, with average accuracies of 93% [54]. However, the average accuracies of other methods were from 67% to 88.7%, suggesting that the batch effects were not soundly addressed. Sarkar et al. [55] selected 17 miRNAs from miRNA expression of 1,707 samples of 10 carefully selected cancer types and 333 normal samples, and normalized miRNA expression in reads per million to $\log_{2}$ scaled values, then 5 classification algorithm achieved average accuracies of 96%.

To overcome these limitations in cancer classification using miRNA sequencing profiles, in this study, we extracted three types of molecular features of miRNAs from 395 small RNA sequencing profiles of LUAD and control samples, i.e., the abundances of original miRNAs (AOM), the abundances of edited miRNAs (AEM) and the editing levels of miRNA editing sites (EL), to construct classification models of LUAD. The Combat-Seq algorithm [56] was used to remove batch effect of the samples. We used eight classification algorithms and achieved very good performances on the combined features including AOM, AEM and EL. We employed three feature selection methods to remove irrelevant and redundant features and compared the performances of the classification algorithms. The DFL algorithm selected three features, i.e., miR-135b-5p, miR-210-3p and miR-182_48u (an edited miRNA), from the training data of 316 samples with combined features. By keeping these three features only, seven algorithms achieved perfect prediction accuracies of 100% on an independent testing data set of 79 samples, which are much better than on AOM features or editing features alone.

## 2 Materials and methods

### 2.1 Small RNA sequencing profiles used

With the informed consent of patients and approval of the Medicine Ethics Committee of The First People's Hospital of Yunnan Province (KHLL2021-KY025), we obtained 19 lung adenocarcinoma tumor and 19 patient-matched non-malignant lung parenchymal tissue samples (used as normal controls) from patients at The First People's Hospital of Yunnan Province, Kunming, Yunnan, China. The obtained tissue samples were put into liquid nitrogen immediately after resection. The total RNAs were retrieved and the small RNA sequencing libraries were prepared by BGI (Shenzhen, China). Then, these small RNA sequencing libraries were sequenced with the BGISEQ-500 sequencer (BGI, Shenzhen, China). The obtained sRNA-Seq profiles were deposited into NCBI GEO database under the series accession number GSE244311.

To increase the diversity and size of the samples, we downloaded another four batches of LUAD and control samples, totally with 357 sRNA-Seq profiles, from the NCBI SRA database with their accession numbers in Table S2. Among these 357 public profiles, 178 were LUAD tissue samples and 179 were paracancerous tissues used as normal control samples (CTL). Totally, 395 sRNA-Seq profiles were analyzed in this study.

### 2.2 Genome sequence and miRNA annotation profiles used

The human unmasked genomic sequences (GRCh38) were downloaded from UCSC Genome Browser [57]. The index of human genomic sequences were generated with the bowtie-build program in the Bowtie package [58]. The sequences of pre-miRNAs and mature miRNAs, and genomic loci of miRNAs in GFF3 format were downloaded from the miRBase (release 21) [59].

### 2.3 Machine learning platform and algorithms used

We used 8 different algorithms to examine the classification of LUAD and control samples based on different types of molecular features of miRNAs. The *k*-Nearest Neighbor (kNN) [60], Decision Tree (C4.5) [61], RandomForest (RF) [62], Support Vector Machine (SVM) [63], and Naive Bayes (NB) [64] algorithms were implemented in WEKA (version 3.8.4) [65], [66], and the eXtreme Gradient Boosting (XGB) [67] and Neural Networks (NN) [68] algorithms were implemented with the Python XGB and PyTorch libraries, respectively. The Discrete Function Learning (DFL) algorithm [69], [70], [71] was also used for feature selection and classification on different miRNA molecular features.

The DFL algorithm is based on information theory. Briefly, the DFL algorithm first identifies a feature subset, **X**, whose mutual information with the class attribute, I(X;Y), equals to the entropy of the class attribute, H(Y) [69], [70], [71]. Based on a theory in Information Theory, the class attribute Y is a function of **X** when I(X;Y)=H(Y). Therefore, the DFL algorithm will construct classification models by removing irrelevant features and combining duplicate (x,y) in the training data [69], [70], [71]. The 1-Nearest-Neighbor algorithm is employed to predict *Y* based on **X** values of testing samples. If a testing sample has the same distance to several (x,y) tuples in the training data, the tuple with largest count value in the training data set will be used to predict *Y* [69], [70], [71]. When the data sets are noisy, I(X;Y)≠H(Y). However, if the part of H(Y) that is not covered by I(X;Y) is very small, the features selected, **X**, are still good to predict the value of *Y* as well [69], [70], [71]. In implementation, if H(Y)−I(X;Y)<ϵ×H(Y), the classification models will be constructed where *ϵ* is a parameter of the DFL algorithm [69], [70], [71].

### 2.4 Identifying microRNA editing sites in LUAD

Firstly, we evaluated the qualities of the sRNA-seq profiles with the FastQC program (https://www.bioinformatics.babraham.ac.uk/projects/fastqc/). If the sequencing score of one or more positions in the first 25 nucleotides (nt) of a sRNA-seq profile was lower than 30, we discarded the profile. If the total number of reads in a sRNA-seq profile is smaller than 1 million, the sRNA-seq profile was discarded too.

The 395 microRNA sequencing profiles selected were then analyzed using the MiRME pipeline [72], [73] with the default settings and parameters. Briefly, the raw reads whose the sequencing scores of the first 25 nucleotides from 5' end had sequencing scores of 30 or higher were kept as qualified reads. Then, we obtained the unique sequences of the remaining reads and calculated the counts of unique reads with more than 18 nucleotides. Next, we aligned the unique reads to human pre-miRNAs using NCBI BLASTN [74] with the options of “-S 1 -m 8 -e 0.01” and the reads mapped to human pre-miRNAs were retrieved. Next, these reads mapped to pre-miRNAs were aligned to the genome using Bowtie (v1.0.0) [75] with the options of “-a -best -S -v 1”. Then, the alignments of reads to genome were examined by the cross-mapping correction method [19] to adjust the weights or percentages of a unique read at each of its genomic loci. In the main step, the MiRME algorithm with the default parameters was used to identify mutation or editing (M/E) sites in miRNAs from the sequences and structures of pre-miRNAs, the alignment of reads to the genome generated by Bowtie, the reads mapped to pre-miRNAs, the alignments of reads to pre-miRNAs generated by BLASTN, and the results of the cross-mapping correction method [19].

The editing level of an M/E site was defined as the number of edited reads over the total number of reads covering the M/E site [72], [73]. The following criteria were used to define editing sites with significant editing levels: (i) the relative editing level was at least 5%; (ii) at least 10 reads supported the editing event; (iii) the score threshold of sequencing reads was 30; and (iv) a multiple-test corrected *P*-value (using the Benjamini and Hochberg method [76]) was smaller than 0.05. Then, the obtained results of different samples were combined by a separate program in the MiRME package (see details in [72], [73]). Only those sites that had significant editing levels in at least one of the samples selected were kept for further analysis.

All identified M/E sites were named by the name of the pre-miRNA, the location of the site, the nucleotides in the reference pre-miRNA sequence, and the edited/mutated nucleotides on the site. The original nucleotide in upper case and the edited/mutated nucleotide in lower case [72], [73]. The edited miRNA corresponding to a miRNA editing site was named with the name of the pre-miRNA, the location of the site, and the edited/mutated nucleotides on the site. For example, hsa-mir-497_25_A_g is an A-to-I editing site on the 25th nucleotide of pre-miR-497, and hsa-mir-497_25g is the edited hsa-miR-197-5p corresponding to this editing site [31].

### 2.5 Generating data sets with diverse miRNA molecular features

A total of 2633 editing or mutation loci were identified by MiRME algorithm. The editing levels of these 2633 M/E sites were extracted from the results of MiRME, then multiplied by 100. Then the average editing levels in the LUAD and CTL group were calculated, respectively. We kept those M/E sites whose editing levels were larger than 10% in either the LUAD or CTL group. The editing levels of 1221 M/E sites were kept for subsequent machine learning analysis (Table S3).

A total of 2568 unique miRNA sequences were obtained from the miRBase. Because the ComBat-seq algorithm [56] in the next step used raw count values, we first calculated the raw counts of the 2568 original miRNAs obtained in the 395 sequencing samples. The sequences of the 2633 mutated/edited miRNAs, corresponding to the 2633 M/E sites, were obtained through MiRME. Then, the raw counts of 2633 edited miRNA sequences in the 395 sequencing samples were calculated. To filter out miRNAs of low expression levels, we kept miRNAs whose mean raw counts in either LUAD samples or normal controls were greater than 10. Totally, 422 original miRNAs and 957 edited or mutated miRNAs (as listed in Table S4 and Table S5, respectively) were kept for further analysis.

Finally, we used three different data sets, the first with 422 AOM (abundances of 422 original miRNAs, as listed in Table S4), the second with 2178 editing features (editing levels of 1221 edited sites and abundances of 957 edited or mutated miRNAs, as listed in Table S6), and the third with 2600 combined features (editing levels of 1221 edited sites, abundances of 422 original miRNAs, abundances of 957 edited or mutated miRNAs, as listed in Table S7), respectively, to evaluate the classification algorithms selected.

### 2.6 Batch effect correction and normalization of the data sets

We first used the prcomp function in the psych package [77] of the R language to perform PCA analysis on the data sets with 422 AOM features, 2178 editing features and 2600 merged features, respectively. Then, we selected the top three principal components with the highest contribution rates in the PCA analysis, and used the plot3 function of MatLab (MathWorks, MA) to visualize the results.

Next, we used the Combat-Seq algorithm [56] to perform batch effect correction for the data sets with 422 AOM features, 2178 editing features and 2600 combined features, respectively. Finally, the data sets after batch effect corrections were normalized using the quantilenorm function in MatLab (MathWorks, MA).

### 2.7 Dividing the data sets into independent training and testing samples

Before performing feature selection and classification, the 395 sequencing samples were randomly divided into training data sets with 80% samples (n = 316) and testing data sets with 20% samples (n = 79). The assignments of samples as training or testing data set were listed in Table S2. To compare the performance of the algorithms on the expression levels of original miRNAs, editing features and combined features of miRNAs, all the data with 422 AOM features, with 2178 editing features and with the 2600 combined features were split into training sets and testing sets with the same samples, respectively. The training sets were used to build the classification models. After that, the testing sets were used to validate the constructed models.

### 2.8 Build the classification models using leave-one-out cross-validation method

Eight classification algorithms selected, i.e., k-Nearest-Neighbors (kNN, k = 6) [78], C4.5 [61], Random Forest (RF) [62], Support Vector Machine (SVM) [79], Naive Bayes (NB) [64], eXtreme Gradient Boosting (XGB) [67], Neural Networks (NN) [68], and Discrete Function Learning (DFL) [69] were used to train classification models using the three different training data sets (n = 316) with 422 AOM features, 2178 editing features and 2600 combined features, respectively. In the process, the leave-one-out cross-validation method was used to set aside one sample of the whole training data sets in a fold to validate the model of the fold. Finally, the average accuracies of the selected algorithms were obtained and compared.

### 2.9 Feature selection and classifier model construction

We used Correlation-based Feature Selection [80], Wrapper for Feature Subset Selection (WFS) [81] implemented in WEKA (version 3.8.4) [66] and DFL [69], [70] for feature selection on training data set (n = 316), respectively. For both CFS and WFS, both the BestFirst and GreedyStepwise algorithm were used as search strategies, respectively. Then, SVM, kNN, RF, C4.5, NB, XGB and NN algorithms were used to construct the classifiers on the feature subsets selected by CFS, WFS, and DFL, respectively.

### 2.10 Validating the classification models with the independent testing data sets

First, the trained classifiers were validated with the testing data set (n = 79) without feature selection, and the accuracies of the eight classification algorithms, i.e., kNN, C4.5, RF, SVM, NB, XGB, NN and DFL were obtained.

Second, the testing data set was trimmed to keep features selected by different feature selection algorithms, i.e., CFS, WFS, and DFL, respectively. Then, seven classification algorithms, i.e., kNN, C4.5, RF, SVM, NB, XGB and NN were evaluated on the trimmed testing data set, respectively. XGB and NN were not applied to WFS features, because WFS should be used with classification algorithms. However, the platforms of XGB/NN and WFS used in this study were different.

### 2.11 Comparing the abundance of original and edited miRNAs

The raw count values of original and edited miRNAs in LUAD and control samples were compared with edgeR [82]. The miRNAs with corrected *P*-values smaller than 0.05 were considered as significantly differently expressed miRNAs in LUAD samples when compared to controls.

### 2.12 Data availability statement

The 38 sRNA-Seq profiles generated in this study are available at the NCBI GEO database through the series accession number GSE244311. Other data sets used in the study are publicly available from the NCBI SRA database with accession numbers listed in Supplementary Table S2.

## 3 Results

### 3.1 sRNA-seq data sets used

We used 395 sRNA-seq profiles of LUAD and controls in this study. Among them, 357 were public profiles, with 178 LUAD and 179 controls, from 4 different studies (see Table S2). Another 38 profiles were generated in this study. Totally, 19 LUAD patients at the First People's Hospital of Yunnan Province, Kunming, Yunnan, China joined the study. The LUAD and control samples of these 19 patients were collected and sequenced by BGI (Shenzhen, China).

In the study, 80% of the 395 samples, i.e., 316 samples (157 LUAD and 159 controls) randomly selected were used as training data set. The 20% remaining samples (with 40 LUAD and 39 controls) were not used in the training processes of the selected algorithms but used as independent testing data set.

### 3.2 MicroRNA molecular features used in machine learning

We used the MiRME pipeline [72] with default parameters to analyze 395 sRNA-seq profiles of LUAD and controls. Totally, we found 2633 significant M/E sites by employing the criteria of at least 10 reads and multiple test corrected *P*-values smaller than 0.05. After removing M/E sites with very limited editing levels, 1221 miRNA editing sites were finally obtained for subsequent analysis (Table S3). In addition, the raw count values of the original and edited miRNAs corresponding to these editing sites were calculated. Then miRNAs with average expression levels of at least 10 raw counts in either the LUAD or the control were kept. Finally, the abundances of 422 original miRNAs (Table S4) and 957 edited or mutated miRNAs (Table S5), respectively, were kept in further analysis.

To demonstrate the advantage of employing the information of miRNA editing/modification in classification, we constructed three different data sets, i.e., the first with 422 AOM features, the second with 2178 editing features (the abundances of 957 edited or mutated miRNAs and editing levels of 1221 miRNA editing sites), and the third with 2600 combined features (the abundances of 422 original miRNAs, abundances of 957 edited or mutated miRNAs, and editing levels of 1221 miRNA editing sites).

### 3.3 PCA analysis and correction of the batch effects

**Figure 1:**
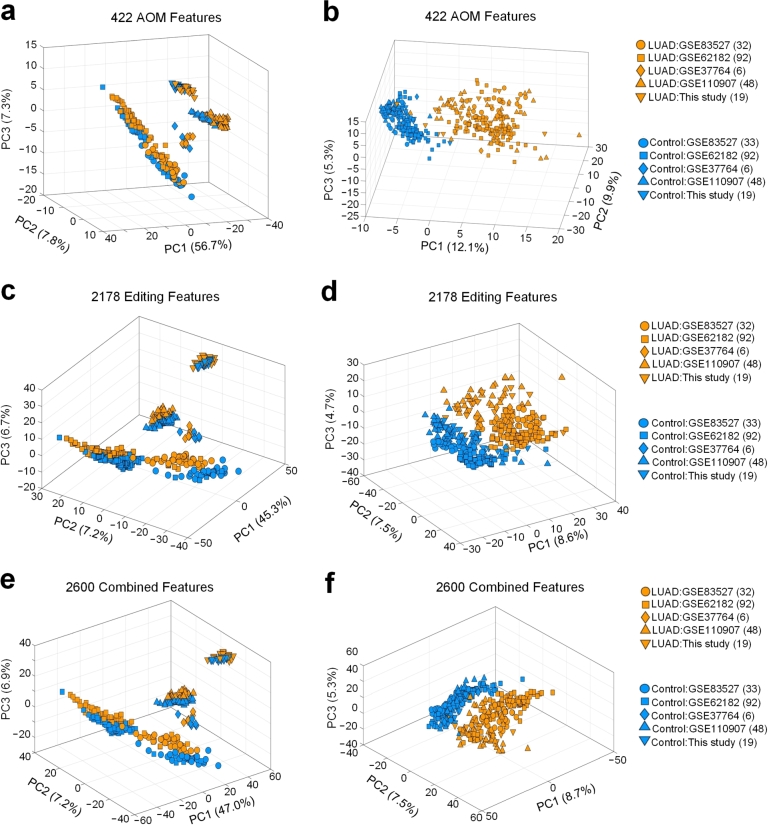
**The PCA (principal component analysis) of the miRNAs data set before and after correction for batch effects.** (**a**) PCA analysis using the 422 AOM features. (**b**) PCA analysis using the 422 AOM features after batch-effect correction with the ComBat-seq algorithm. (**c**) PCA analysis using the 2178 editing features. (**d**) PCA analysis using the 2178 editing features after batch-effect correction with the ComBat-seq algorithm. (**e**) PCA analysis using the 2600 combined miRNA features. (**f**) PCA analysis using the 2600 combined miRNA features after batch-effect correction with the ComBat-seq algorithm. See also Table S11 for source data.

PCA analysis was performed on 422 AOM features, 2178 editing features and 2600 combined features, respectively, as shown in Fig. 1a, 1c and 1e, respectively. Very obvious sample batch effects are witnessed on all data sets (see Fig. 1a, 1c and 1e, respectively) because the samples from the five different studies are clustered together, and clusters of different studies locate in different positions in the PCA plots. These results indicate that the data in the 5 different studies have strong between-study differences. As shown in Fig. 1a, 1c and 1e, the LUAD (yellow markers) and control samples (blue markers) have clear differences in the data sets of the five studies used, respectively.

In order to eliminate the influence of batch effects, the Combat-seq algorithm [56] was used to correct the batch effect of the data sets used. Then, quantilenorm was used to normalize the abundances of miRNAs and editing levels of M/E sites. To show the effect of batch effect correction, we performed PCA analysis again on the data after batch effect correction. As shown in Fig. 1b, 1d and 1f, the LUAD samples and control samples of the five studies are merged and grouped into two different clusters, respectively. This indicates that the selected ComBat-seq algorithm successfully corrects the batch effects in different studies.

### 3.4 Evaluating the performances of the selected algorithms on the AOM, editing and combined features

We first applied eight classification algorithms, i.e., kNN, C4.5, RF, SVM, NB, XGB, NN and DFL, on the training data set with the leave-one-out cross-validation. The obtained classifiers were tested using the independent test data set, and the accuracies of the classifiers were obtained (Table 1). On 422 AOM features, the accuracies of the eight algorithms are 96.2%, 96.2%, 100%, 97.5% 94.9%, 98.7%, 96.2% and 93.7%, respectively on the testing data set with 422 AOM features. On the editing features, the accuracies of the eight algorithms are 89.9%, 93.7%, 97.5%, 96.2%, 94.9%, 98.7%, 93.7% and 97.5%, respectively. On the combined features, the accuracies of the eight algorithms are 94.9%, 97.5%, 98.7%, 94.9%, 94.9%, 100%, 97.5% and 100%, respectively. The RF algorithm had the best performance on the 422 AOM features, and the XGB and DFL algorithm performed the best on the combined features, both of these three algorithms reached 100% accuracy.

**Table 1:** The accuracies of the selected algorithms on the 422 AOM features, 2178 editing features and 2600 combined features, respectively. The unit is percent.

Features	*Classification Algorithm*							
	kNN	C4.5	RF	SVM	NB	XGB	NN	DFL
*422 AOM features*								
*LOOCV* (Training, n = 316)	96.2	93.0	98.4	98.1	93.0	**98.7**	88.3	94.6
Training (n = 316) vs Testing (n = 79)	96.2	96.2	**100**	97.5	94.9	98.7	96.2	93.7

*2178 editing features*								
*LOOCV* (Training, n = 316)	95.9	92.4	97.8	97.8	93.7	**98.1**	94.3	96.5
Training (n = 316) vs Testing (n = 79)	89.9	93.7	97.5	96.2	94.9	**98.7**	93.7	97.5

*2600 combined features*								
*LOOCV* (Training, n = 316)	95.3	96.2	97.8	98.1	94.6	**98.7**	94.6	98.1
Training (n = 316) vs Testing (n = 79)	94.9	97.5	98.7	94.9	94.9	**100**	97.5	**100**

**Abbreviations:** kNN, k-Nearest Neighbors algorithm; C4.5, C4.5 Decision Tree algorithm; RF, Random Forest algorithm; SVM, Support Vector Machine algorithm; NB, Naive Bayes; XGB, eXtreme Gradient Boosting; NN, Neural Networks; DFL, Discrete Function Learning algorithm.

When being compared with results on AOM features, the performances of the 8 classification algorithms on the 2178 editing features were also very good. NB and XGB had the same accuracies on 2178 editing features as on 422 AOM features. DFL had a better prediction performance on the editing features than on AOM features. As shown in Table 1, C4.5, XGB and DFL performed better on 2600 combined features than on 422 AOM features, and kNN, C4.5, RF, XGB, NN and DFL performed better on 2600 combined features than on 2178 editing features. Five algorithms, i.e., kNN, RF, SVM, NB and NN had slightly worse or equal prediction performances on the 2600 combined features than on 422 AOM features, suggesting possible irrelevant or redundant features in the 2600 combined features.

### 3.5 The results of feature selection

**Figure 2:**
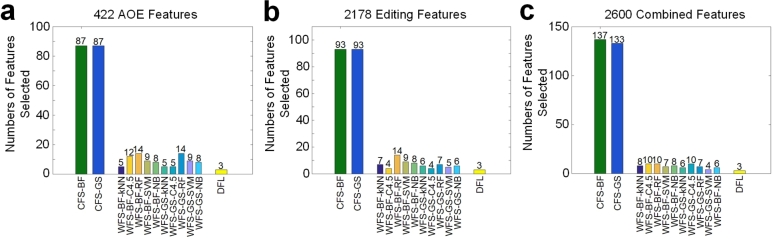
**The numbers of features selected by different feature selection algorithms.** (**a**) The numbers of features selected by different feature selection algorithms from the 422 AOM features. (**b**) The numbers of features selected by different feature selection algorithms from the 2178 editing features. (**c**) The numbers of features selected by different feature selection algorithms from 2600 combined features.

Because there could be irrelevant and redundant features in the data sets, we used three feature selection methods, Correlation-based Feature Selection (CFS) [80], Wrapper for Feature Subset Selection (WFS) [81] implemented in WEKA [66] and DFL [69], [70], respectively, to perform feature selection on the training set data. For the data with 422 AOM features, the CFS algorithms selected 87 feature, the WFS algorithm selected 5 to 14 features when being used with different classification algorithms, and the DFL algorithm only selected 3 features (Fig. 2a). For the data with 2178 editing features, the CFS selected 93 features, the WFS algorithm selected 4 to 14 features when being used with different classification algorithms, and the DFL algorithm also selected 3 features (Fig. 2b). For the data with 2600 combined features, the CFS selected over 130 features, the WFS algorithm selected 4 to 10 features when being used with different classification algorithms, and the DFL algorithm also selected 3 features (Fig. 2c). The features selected by DFL are hsa-miR-183-5p, hsa-miR-218-5p and hsa-miR-6510-3p for the data set with 422 AOM features (Table S8), hsa-mir-30a_68a, hsa-mir-139_30_G_a, hsa-mir-210_88a for the data set with 2178 editing features (Table S9) and hsa-miR-135b-5p, hsa-miR-210-3p and hsa-miR-182_48u for the data sets with 2600 combined features (Table S10).

### 3.6 The ROC curves of the model corresponding to the subset of features

**Figure 3:**
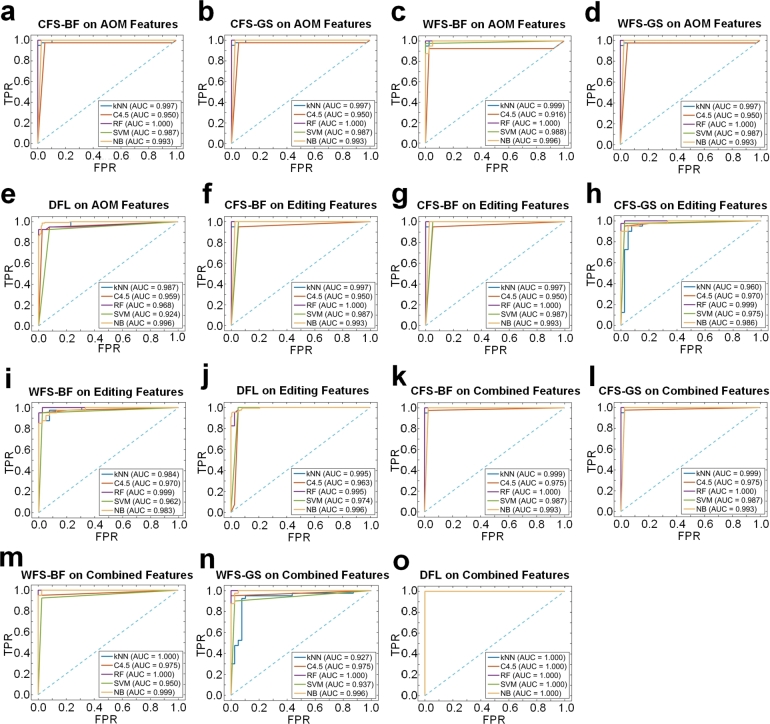
**The ROC curves when using different sets of features.** (**a**) The AOM features selected by the CFS algorithm with the BestFirst for search. (**b**) The AOM features selected by the CFS algorithm with the GreedyStepwise for search. (**c**) The AOM features selected by the WFS algorithm with the BestFirst for search. (**d**) The AOM features selected by the WFS algorithm with the GreedyStepwise for search. (**e**) The AOM features selected by the DFL algorithm. (**f**) The features selected from 2178 editing features by the CFS algorithm with the BestFirst for search. (**g**) The features selected from 2178 editing features by the CFS algorithm with the GreedyStepwise for search. (**h**) The features selected from 2178 editing features by the WFS algorithm with the BestFirst for search. (**i**) The features selected from 2178 editing features by the WFS algorithm with the GreedyStepwise for search. (**j**) The features selected from 2178 editing features by the DFL algorithm. (**k**) The features selected from 2600 combined features by the CFS algorithm with the BestFirst for search. (**l**) The features selected from 2600 combined features by the CFS algorithm with the GreedyStepwise for search. (**m**) The features selected from 2600 combined features by the WFS algorithm with the BestFirst for search. (**n**) The features selected from 2600 combined features by the WFS algorithm with the GreedyStepwise for search. (**o**) The features selected from 2600 combined features by the DFL algorithm. See also Table S12 for source data.

We used ROC curves to evaluate the performance of different machine learning algorithms on features selected by different feature selection methods. As shown in Fig. 3a to 3e, the AUC values of the four algorithms, i.e., kNN, C4.5, RF and SVM, for different AOM feature subsets are all above 90%, where the lowest value is 91.6% by the C4.5 algorithm using with WFS on the 422 AOM features. The AUC value of the RF algorithm is 100% for the AOM features selected by CFS and WFS, and is 96.8% for the 3 AOM selected by DFL (Fig. 3e). As shown in Fig. 3f to 3j, the lowest AUC value for different editing features subsets is 95% by the C4.5 algorithm using with CFS. The AUC value could reach 100% by the RF algorithm for the editing features selected by CFS. Furthermore, when comparing the AUC values on the 422 AOM features and 2178 editing features with 2600 combined features (Fig. 3k to 3o), respectively, the AUC values of the four classification algorithms indeed increase on data sets with 2600 combined features, suggesting that the additional information of miRNA editing and/or modification events, i.e., the editing levels and abundances of edited miRNAs, is helpful in improving the classification performances.

### 3.7 Evaluating the classifiers on the selected features

We trimmed the independent testing data to keep features selected by different feature selection methods, and the trimmed data sets were used to evaluate the performances of different algorithms. As shown in Table 2, the RF algorithm reached 100% prediction accuracy on miRNA abundance features selected by CFS and WFS. All of the 7 algorithms performed better on the editing features selected by DFL than on AOM features selected on DFL. The performances of the 7 algorithms were slightly worse on the editing features selected by CFS and WFS compared to on AOM features selected by CFS and WFS. The kNN, C4.5 and XGB algorithm had better performances on the data sets of 2600 combined features than on 422 AOM features and on 2178 editing features. Seven classification algorithms reached 100% prediction accuracies on the features selected by the DFL algorithm for the data set with 2600 combined features, which were much better than those on the data of 422 AOM features and 2178 editing features. In summary, these again indicate that the editing levels of miRNA M/E sites and abundances of edited miRNAs are useful in improving the classification of LUAD samples.

**Table 2:** The accuracies of five classification algorithms on features selected by different feature selection methods. The column of “No. of Features” listed the numbers of the features selected by the feature selection algorithm in the same line.

Feature Selection Method	No. of Features	*Classification Algorithm*						
		kNN	C4.5	RF	SVM	NB	XGB	NN
*422 AOM features*								
CFS + BestFirst	87	96.2	96.2	**100**	98.7	**100**	98.7	98.7
CFS + GreedyStepwise	87	96.3	96.3	**100**	98.7	**100**	98.7	**100**
Wrapper + BestFirst	5 to 14	97.5	94.9	**100**	98.7	94.9	NA	NA
Wrapper + GreedyStepwise	5 to 14	97.5	94.9	**100**	98.7	94.9	NA	NA
DFL	3	93.7	93.7	93.7	92.4	93.7	93.7	96.2

*2178 editing features*								
CFS + BestFirst	93	97.5	94.9	98.7	97.5	97.5	98.7	**100**
CFS + GreedyStepwise	93	97.5	94.9	98.7	97.5	97.5	98.7	**100**
Wrapper + BestFirst	4 to 14	92.4	96.2	98.7	97.5	93.7	NA	NA
Wrapper + GreedyStepwise	4 to 7	93.7	96.2	96.2	96.2	93.7	NA	NA
DFL	3	97.5	97.5	97.5	97.5	98.7	98.7	96.2

*2600 combined features*								
CFS + BestFirst	137	98.7	97.5	98.7	98.7	98.7	**100**	98.7
CFS + GreedyStepwise	133	98.7	97.5	98.7	98.7	98.7	**100**	98.7
Wrapper + BestFirst	7 to 10	**100**	97.5	98.7	94.9	96.2	NA	NA
Wrapper + GreedyStepwise	4 to 7	92.4	97.5	98.7	93.7	97.5	NA	NA
DFL	3	**100**	**100**	**100**	**100**	**100**	**100**	**100**

**Abbreviations:** kNN, k-Nearest Neighbors algorithm; C4.5, C4.5 Decision Tree algorithm; RF, Random Forest algorithm; SVM, Support Vector Machine algorithm; NB, Naive Bayes; XGB, eXtreme Gradient Boosting; NN, Neural Networks; DFL, Discrete Function Learning algorithm.

When comparing the results in Table 2 to those in Table 1, it is shown that the feature selection improve the performances of the four classification algorithms. Particularly, all of the five classification algorithms achieved perfect accuracies of 100% when they were applied to 3 features selected from 2600 combined features by the DFL algorithm.

### 3.8 Examining features selected by DFL algorithm

**Figure 4:**
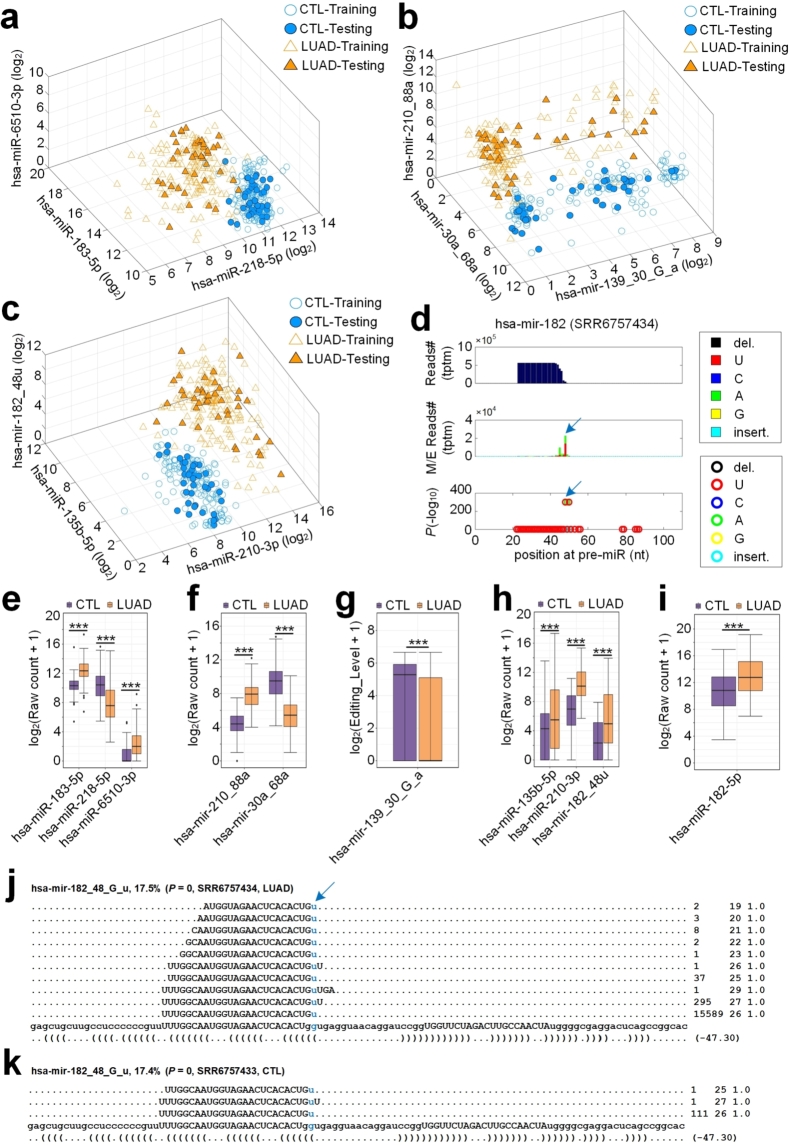
**The distributions of samples in the feature spaces selected by the DFL algorithm.** (**a**) The distribution of samples in the space of the three AOM features selected by DFL. (**b**) The distribution of samples in the space of the three features selected from 2178 editing features by DFL. (**c**) The distribution of samples in the space of the three features selected from 2600 combined features by DFL. (**d**) The MiRME map of hsa-miR-182 in one of the LUAD samples (SRR6757434) (**e**) Comparisons of abundances of hsa-miR-183-5p, hsa-miR-218-5p and hsa-miR-6510-3p in LUAD and CTL samples. (**f**) Comparisons of abundances of hsa-mir-210_88a and hsa-mir-30a_68a in LUAD and CTL samples. (**g**) Comparisons of the editing level of hsa-mir-139_30_G_a in LUAD and CTL samples. “***” indicates *P*-value smaller than 0.001, Mann-Whitney *U*-test. (**h**) Comparisons of abundances of hsa-miR-135b-5p, hsa-miR-210-3p and hsa-mir-182_48u in LUAD and CTL samples. (**i**) Comparisons of abundances of hsa-miR-182-5p in LUAD and CTL samples. (**j**) The details of hsa-mir-182_48_G_u in one of the LUAD samples (SRR6757434). (**k**) The details of hsa-mir-182_48_G_u in one of the normal control samples (SRR6757433). In Part (**e**), (**f**), (**h**) and (**i**), “***” indicates corrected *P*-values (with the Benjamini and Hochberg method [76]) smaller than 0.001, exact negative Binomial tests implemented in edgeR [82]. See also Table S13 for source data.

The DFL algorithm selected 3 miRNA features, i.e., hsa-miR-183-5p, hsa-miR-218-5p and hsa-miR-6510-3p, from the 422 AOM features; selected 3 miRNA features, i.e., hsa-mir-30a_68a, hsa-mir-139_30_G_a, hsa-mir-210_88a, from the 2178 editing features; and selected 3 miRNA features, i.e., hsa-miR-135b-5p, hsa-miR-210-3p and hsa-miR-182_48u from 2600 combined features, respectively. To show the value of these features, we plotted the samples in the spaces defined by these three sets of features in Fig. 4a, 4b and Fig. 4c, respectively. As shown in Fig. 4a, most LUAD samples located on the left of the space, and most control samples located on the right side of the space. As shown in Fig. 4b, some LUAD samples mixed with control samples on the left of space. As shown in Fig. 4c, the difference between the LUAD and control samples were much clearer than those in Fig. 4a and 4b, indicating that the editing information of hsa-miR-182 is very helpful in enlarging the gap between LUAD and control samples. This is consistent with better performances of the classification algorithms when applied to data set with hsa-miR-135b-5p, hsa-miR-210-3p and hsa-miR-182_48u than to that with hsa-miR-183-5p, hsa-miR-218-5p and hsa-miR-6510-3p, and to that with hsa-mir-30a_68a, hsa-mir-139_30_G_a, hsa-mir-210_88a.

As shown in Fig. 4e, hsa-miR-183-5p, and hsa-miR-6510-3p had significantly higher expression levels (corrected P<0.05, edgeR) in LUAD samples than in normal control samples, but hsa-miR-218-5p had significantly lower expression level (corrected P<0.05, edgeR) in LUAD samples than in normal controls. Two edited miRNAs, i.e., hsa-mir-210_88a and hsa-mir-30a_68a, are up- and down-regulated in LUAD samples compared to controls (Fig. 4f), respectively. The editing level of hsa-mir-139_30_G_a is significantly lower in LUAD than in controls (Fig. 4g). As shown in Fig. 4h, hsa-miR-135b-5p, hsa-miR-210-3p and hsa-miR-182_48u were universally significantly upregulated (corrected P<0.05, edgeR) in LUAD samples when compared to controls. hsa-miR-182-5p was upregulated in LUAD compared to normal controls (Fig. 4i).

As shown in Fig. 4d, hsa-miR-182a_48u is generated by an 3'-U events at the end of hsa-miR-182-5p. Fig. 4j and 4k shows the details of hsa-miR-182_48_G_u in one of the LUAD samples (SRR6757434) and one of the control samples (SRR6757433). As shown in Fig. 4j and 4k, the edited hsa-miR-182-5p, i.e., hsa-miR-182a_48u, is formed by adding a uracil at the 3' end of hsa-miR-182-5p, i.e., UUUGGCAAUGGUAGAACUCACACUGu. By comparing Fig. 4j and 4k, hsa-miR-182a_48u had much higher expression level in the LUAD sample selected (SRR6757434) than in the control sample selected (SRR6757433).

## 4 Discussion

In this study, we used the several different types of molecular features of miRNAs, including the abundances of original miRNAs, the abundances of edited miRNAs, and the editing levels of miRNA M/E sites, to classify LUAD samples. We found that seven classification algorithms i.e., kNN, C4.5, RF, SVM, NB, XGB and NN, could reach perfect prediction accuracies of 100% on three miRNA features, i.e., hsa-miR-135b-5p, hsa-miR-210-3p and hsa-miR-182_48u, selected by the DFL algorithm on an independent testing data set with 79 samples. As a comparison, we used the DFL algorithm to select the abundances of three original miRNAs, i.e., hsa-miR-183-5p, hsa-miR-218-5p and hsa-miR-6510-3p, from a separate data set of original miRNA abundance values. The seven classification algorithms selected had much lower classification accuracies, 92.4% to 96.2%, on these three original miRNA features for the independent testing set. Furthermore, we also used the DFL algorithm to select three editing features, i.e., hsa-mir-30a_68a, hsa-mir-139_30_G_a and hsa-mir-210_88a, as parallel experiments. On these three editing features selected by DFL, the seven classification algorithms could achieve accuracies 96.2% to 98.7%, which were lower than those obtained from combined features too. In summary, different classification algorithms achieved better performances when being applied to combined miRNA features, i.e., abundances of original and edited miRNAs and editing levels of miRNA editing sites, than on abundances of original miRNAs or editing features of miRNAs alone. These results indicate that the editing features of miRNAs are good supplements to the abundances of miRNAs in the classification of LUAD and control samples.

Our results showed that performances of the 7 classification algorithms were very good on the features selected by the DFL algorithm (as shown in Table 2). There are two potential advantages of using less features in performing classification. Firstly, the less numbers of features chosen by the DFL algorithm make the classification models simpler than those with more features chosen by CFS or WFS. Based on the Occam's razor, the simpler models are preferred over more complex ones, since they have lower risks of over-fitting the training data sets if the simpler models can achieve comparable performances to those of more complex models. Secondly, smaller number of features also makes it easier to employ the selected miRNAs in clinical applications, such as designing new diagnostic methods of LUAD based on these miRNAs.

Recently, Distefano et al. [51] employed the abundances of original and edited miRNAs (i.e., isomiRs) to improve clustering of cancer samples. The integration of edited miRNAs also figured out unique clinicopathologic features among cohorts [51]. Our results suggest that editing levels of miRNA editing sites are also useful in classification of cancer samples, in addition to the abundances of original and edited miRNAs.

We carefully reviewed literature to find the functional relevances for the miRNAs identified by the DFL algorithm in LUAD, and found that hsa-miR-183-5p, hsa-miR-218-5p, hsa-miR-6510-3p, hsa-miR-135b-5p, hsa-miR-210-3p and hsa-miR-182 were reported to have clear roles in LUAD [83], [84], [85], [86], [87], [88]. Wang et al. reported that hsa-miR-183-5p was significantly upregulated in LUAD and hsa-miR-183-5p could promote lung carcinogenesis by directly targeting phosphatase tensin (PTEN), suggesting that hsa-miR-183-5p is a promising target in the diagnosis and treatment of lung cancer [83]. In 2022, Chen et al. revealed that hsa-miR-218-5p was significantly downregulated in LUAD, and it could interact with 3'-untranslated region (UTR) of ERO1A mRNA, which enables the ability of hsa-miR-218-5p to restrain cell viability, invasion, and migration in LUAD [84]. Fan et al. identified that hsa-miR-6510-3p was a differentially expressed autophagy-related miRNA between radiotherapy-resistent and radiotherapy-sensitive groups of Non-small-cell lung cancer(NSCLC) patients in 2021, and the expression of hsa-miR-6510-3p was upregulated in radiotherapy-sensitive group, becoming the foundation for further research on insensitivity of radiotherapy of NSCLC patients [85].

In 2021, Zhao et al. [86] demonstrated that hsa-miR-135b-5p was markedly upregulated in LUAD and directly targeted the 3'-UTR of the deubiquitinase CYLD, thereby modulating ubiquitination and activation of NF-*κ*B signaling. Interleukin-6 (IL-6)/STAT3 could elevate miR-135b-5p level and that STAT3 directly bound the promoter of hsa-miR-135b-5p, a new positive feedback loop of the IL-6/STAT3/miR-135b/NF-*κ*B was found in NSCLC and hsa-miR-135b-5p could be a potential therapeutic target for NSCLC [86]. In 2021, Chen et al. [87] proved that hsa-miR-210-3p was upregulated in lung cancer tissues, and hsa-miR-210-3p facilitated lung cancer development and metastasis by impairing USF1-mediated promotion of PCGF3. This provided a novel understanding of the mechanism of lung cancer development and metastasis [87]. In 2021, Yang et al. [88] demonstrated that hsa-miR-182-5p is significantly upregulated in LUAD and regulates lung adenocarcinoma metastasis and epithelial-mesenchymal transition by targeting EPAS1.

The 3' ends of mature miRNAs are highly heterogeneous, often containing 1-3 extra nucleotides that do not match the genomic DNA sequences. These un-templated nucleotides are added by terminal nucleotidyl transferases that preferentially introduce uridyl or adenyl residues to the 3' terminus of RNAs [32]. Terminal Uridylyl Transferases (TUT) 4 and 7 are the enzymes responsible for 3' uridylation of miRNAs [36], [32]. Several studies reported that TUT4/7 may either add oligouridine tail to pre-let-7 to suppress Dicer processing [89] or add one uracil to enhance Dicer processing [33]. As shown in Fig. 4g and 4h, hsa-mir-182_48u is formed by adding a uracil base at the 3' end of hsa-miR-182-5p. As noticed previously [35], [72], the 3'-U or 3'-A of miRNAs at 5' arms of pre-miRNAs may happen after the loops of pre-miRNAs are cleaved by Dicer. The expression level of hsa-miR-182-5p is increased in LUAD samples when compared to controls (see Fig. 4e). More studies are expected to clarify the relation between the 3'-U addition of hsa-miR-182-5p and the expression of hsa-miR-182-5p. As reported by Yang et al. [90], uridylated miR-27a gains a set of non-canonical targets. Therefore, it is interesting to examine whether uridylated hsa-miR-182-5p also acquires some novel targets and plays a role in the initiation and progress of LUAD.

Because seven classification algorithms perform excellently on the three features, i.e., hsa-miR-135b-5p, hsa-miR-210-3p and hsa-miR-182_48u, it may also be feasible to employ these miRNAs as biomarkers for diagnosis of LUAD.

## 5 Conclusions

Because the LUAD samples used in this study were obtained from five different researches, and the small RNA sequencing profiles were generated by different sequencing platforms, the batch effects and systematic differences within the samples should be removed. We employed the ComBat-seq algorithm [56] to remove the batch effect, and used quantilnorm to normalize the raw counts of miRNAs and editing levels of miRNA M/E sites. These steps successfully removed the batch effects in the samples selected. Our results show that on the three features, i.e., hsa-miR-135b-5p, hsa-miR-210-3p and hsa-miR-182_48u selected by the DFL algorithm, seven classification algorithms achieve perfect classification accuracies of 100%, which are better than on miRNA abundances or miRNA editing features alone. These results demonstrate that classification performances of LUAD are improved by integrating information of miRNA editing.

## Funding

The research was supported in part by a grant (No. 31760314) of 10.13039/501100001809National Natural Science Foundation of China (http://www.nsfc.gov.cn/), and a grant (No. SKLGE-2107) of the Open Research Funds of the 10.13039/501100011211State Key Laboratory of Genetic Engineering, Fudan University, China, to YZ. The funders had no role in study design, data collection and analysis, decision to publish, or preparation of the manuscript.

## CRediT authorship contribution statement

**Shiyong Guo:** Writing – original draft, Validation, Investigation, Formal analysis, Data curation. **Chunyi Mao:** Writing – review & editing, Visualization, Investigation, Formal analysis, Data curation. **Jun Peng:** Resources. **Shaohui Xie:** Software, Methodology. **Jun Yang:** Investigation, Formal analysis. **Wenping Xie:** Investigation, Formal analysis. **Wanran Li:** Investigation, Formal analysis. **Huaide Yang:** Investigation, Formal analysis. **Hao Guo:** Investigation, Formal analysis. **Zexuan Zhu:** Software, Methodology. **Yun Zheng:** Writing – review & editing, Visualization, Supervision, Software, Project administration, Methodology, Investigation, Funding acquisition, Conceptualization.

## Declaration of Competing Interest

The authors declare that they have no known competing financial interests or personal relationships that could have appeared to influence the work reported in this paper.

## Appendix A Supplementary material

MMC 1 The accuracies of classification models in other published studies using miRNA expression. MMC 1

MMC 2 The information of 395 small RNA sequence profiles. MMC 2

MMC 3 The editing levels of 1221 editing sites in the 395 samples selected. MMC 3

MMC 4 The abundances (raw count) of 422 original miRNAs in the 395 samples selected. MMC 4

MMC 5 The abundances (raw counts) of 957 mutated and/or edited miRNAs in the 395 samples selected. MMC 5

MMC 6 The values of 2178 editing features in the 395 samples selected. MMC 6

MMC 7 The values of 2600 combined miRNA features in the 395 samples selected. MMC 7

MMC 8 The features selected by different feature selection methods from the abundances of original miRNAs. MMC 8

MMC 9 The features selected by different feature selection methods from the editing features. MMC 9

MMC 10 The features selected by different feature selection method from the combined features. MMC 10

MMC 11 The source data of Figure 1. MMC 11

MMC 12 The source data of Figure 3. MMC 12

MMC 13 The source data of Figure 4. MMC 13
